# Detection and Classification of Root and Butt-Rot (RBR) in Stumps of Norway Spruce Using RGB Images and Machine Learning

**DOI:** 10.3390/s19071579

**Published:** 2019-04-01

**Authors:** Ahmad Ostovar, Bruce Talbot, Stefano Puliti, Rasmus Astrup, Ola Ringdahl

**Affiliations:** 1 Division of Forestry and Forest Resources, Norwegian Institute of Bioeconomy Research (NIBIO), P.O. Box 115, 1431 Ås, Norway; Bruce.Talbot@nibio.no (B.T.); Stefano.Puliti@nibio.no (S.P.); rasmus.astrup@nibio.no (R.A.); 2 Department of Computing Science, Umeå University, 901 87 Umeå, Sweden; ringdahl@cs.umu.se

**Keywords:** deep learning, forest harvesting, tree stumps, automatic detection and classification

## Abstract

Root and butt-rot (RBR) has a significant impact on both the material and economic outcome of timber harvesting, and therewith on the individual forest owner and collectively on the forest and wood processing industries. An accurate recording of the presence of RBR during timber harvesting would enable a mapping of the location and extent of the problem, providing a basis for evaluating spread in a climate anticipated to enhance pathogenic growth in the future. Therefore, a system to automatically identify and detect the presence of RBR would constitute an important contribution to addressing the problem without increasing workload complexity for the machine operator. In this study, we developed and evaluated an approach based on RGB images to automatically detect tree stumps and classify them as to the absence or presence of rot. Furthermore, since knowledge of the extent of RBR is valuable in categorizing logs, we also classify stumps into three classes of infestation; rot = 0%, 0% < rot < 50% and rot ≥ 50%. In this work we used deep-learning approaches and conventional machine-learning algorithms for detection and classification tasks. The results showed that tree stumps were detected with precision rate of 95% and recall of 80%. Using only the correct output (TP) of the stump detector, stumps without and with RBR were correctly classified with accuracy of 83.5% and 77.5%, respectively. Classifying rot into three classes resulted in 79.4%, 72.4%, and 74.1% accuracy for stumps with rot = 0%, 0% < rot < 50%, and rot ≥ 50%, respectively. With some modifications, the developed algorithm could be used either during the harvesting operation to detect RBR regions on the tree stumps or as an RBR detector for post-harvest assessment of tree stumps and logs.

## 1. Introduction

The annual losses due to conifer root and butt-rot (RBR) caused by fungi in the Heterobasidion genus is estimated to amount to some 800 million € in Europe [1]. Both Scots pine (Pinus sylvestris) and Norway spruce (Picea abies) are affected by Heterobasidion species, but unlike in pine, in Norway spruce the decay columns can rise up the stem to a height of 10–12 m. This makes the infection particularly damaging in Norway spruce forests, as it destroys the most valuable part of the tree, reducing it from saw log quality to lower value pulp or energy wood [2]. A Norwegian decay survey showed that in managed spruce forests, on average every 5th harvested tree showed signs of stem decay caused by Heterobasidion [3]. A major challenge for reducing RBR is that Heterobasidion remains infectious in stumps and root systems of diseased trees for decades, which efficiently transfers the disease to the next forest generation (e.g., [4,5,6]). However, to date the lack of site-specific maps on the occurrence of decay has prevented the implementation of best-practice management.

In forestry, the amount of site-specific information generated from various emerging and existing technologies is rapidly increasing. Timber harvesters store comprehensive forest information through the Standard for Forest machine Data and Communication (StanForD) recording system [7]. In principle, the StanForD data can be supplemented by operator inputs [8] or through additional machine-mounted sensors [9,10] to observe and record the surrounding environment in greater detail. Technological advancements are making it possible to estimate the actual position of the harvesting head and thus of the individual harvested tree with high precision [11]. Currently, harvesting machine operators visually detect the presence of RBR through a change in color of the sawdust ejected during the felling cut, after which they will move the crane boom in order to be able to make a visual assessment of the extent of rot before cutting the tree to various log assortments. However, timber harvester operators experience a high mental workload with up to 4000 control inputs per hour [12]. To derive objective and cost-effective measures, it would be beneficial to develop methods to automatically detect the presence of RBR from images taken at the time of felling. Machine vision is already gaining ground in forest operations by providing autonomous detection [13,14] and automating decision making with the aim of relieving the cognitive workload on the operators, e.g., [15]. A real-time detection of rot would further enable computer assisted qualitative bucking of logs to products.

The importance of RBR detection and localization in forestry is well known, and numerous technologies ranging from increment borers, through electrical conductivity, ultrasonic detectors, thermography, and computed tomography have been demonstrated [16]; however, few mobile automated systems have been applied for this purpose. Recently with increasing usage of unmanned aerial vehicles (UAVs) in forestry, researchers developed approaches to automatically detect stumps using aerial images in post-harvest sites [17,18]. Samiappan, et al. [17] automatically detected stumps and measured their diameter in aerial images using image pattern recognition techniques (i.e., Template Matching, Hough Circle Transform, and Phase Coding) to detect circular patterns [19], calculate tree-stump diameter, and compare their performance. Puliti, et al. [18] developed a stump detection and segmentation algorithm using spectral and three-dimensional UAV photogrammetric data. In their method, after an initial seed points definition according a local maxima search, the stumps were segmented and classified according to the presence of RBR. Their results were encouraging but also highlighted some limitations of using UAVs in mapping RBR. In particular, their results revealed that it was possible to accurately assess only those stumps that were not occluded by logging residues or altered by forestry machines driving over them. In this respect, the development of a method to detect and classify the stumps in real time right after the tree is felled would be beneficial as it would allow for a full census of the tree stumps, rather than an unknown sample size.

Rot detection is a difficult task because it does not have many characteristic properties. Furthermore, existing defect detection systems were developed for use in sawmills, where the environmental conditions such as lighting conditions and occlusions are controlled and the task is limited to detecting defects such as knots or discoloration on the sawn wooden boards [20,21,22,23]. Since each piece of wood has unique feature characteristics, applying machine vision techniques to wood products processes involves a complicated set of element modification for each application [24]. To achieve reliable defect detection several studies have been conducted using various types of sensors. Methods developed for defect detection based on visual spectrum include the use of RGB cameras and spectrometers. The types of algorithms used were based on image segmentation methods and can be divided [25] into histogram thresholding, region-based approaches, feature space clustering, edge detection, neural networks, fuzzy approaches, physics-based approaches, and any combination of the mentioned techniques. Estevez et al. [22] performed image segmentation by histogram-based multiple thresholding and achieved 95% accuracy; however, the improvements in accuracy came at the cost of increased rate of false positives. In [23,26] a method for wood surface defect detection called fuzzy min-max neural network [27] was developed for image segmentation (FMMIS). They used a neurofuzzy color image segmentation method to generate seed points and then used the FMMIS method to grow boxes from these points, to find the minimum bounding boxes for each defect present in the wood board image. Duncker and Spiecker [28] developed a methodology based on reflected light to detect compression wood in a stem cross section of Norway spruce. Cross sections are classified using Spectral Angle Mapper algorithm, by comparing the standardized spectrum of each pixel with reference spectra stored in a spectral library. Using edge detection methods for defect detection, Poelzleitner [20] used the Hough transformation to identify and classify defects while Lepage et al. [29] used a pyramidal multi-resolution approach. Zhong [21] developed a multi-thresholding extension of Otsu [30] to detect defects on timbers. Puliti, et al. [18] also extracted 17 features from orthophoto images of UAV-detected stumps and used a random forest classifier to detect RBR in stumps and obtained an overall classification accuracy for presence or absence of RBR of 80%. It should be stressed that apart from [18], all the defect detection algorithms are applied on sawn timber and in a controlled environment.

The motivation behind the present study is that timber harvesters could ultimately be enabled to autonomously collect information on the presence and severity of RBR. This could be used in generating both site-level and national maps of the occurrence of rot. The availability of such maps could also help to shed new light on the biology behind the spread of RBR within single trees and among tree communities. Furthermore, these same maps could be used as ground reference data for training large-scale remote sensing-based models of RBR. The specific aim of this study was therefore to develop a novel approach for tree-stump and RBR detection and severity assessment from close-range single stump images using deep-learning algorithms. These images were taken in such a way to simulate what could be acquired from a harvester head or crane boom. The automated proposed method was developed with particular attention to being independent from subjective parameter tuning, thus generally applicable.

Since the aim of this work was to detect the existence of RBR on tree stumps, it was necessary to first detect the stumps in the images. Object detection in forestry is challenging due to factors such as dynamic illumination conditions, occlusion, and an unstructured environment. The most common approaches for object detection in forestry is using different methods of thresholding, such as multi-thresholding, adaptive thresholding, and seed point generation based on thresholds, as well as conventional machine-learning algorithms. A weakness with these approaches is the need to hand-tune parameters, which results in them performing less accurately in similar tasks with different conditions. Despite the fact that there has been a great interest in deep-learning-based models due to their success in object detection competitions such as ImageNet Large-Scale Visual Recognition Challenge (ILSVRC) [31] and MS-COCO [32], to the best of the authors’ knowledge, none of the state of the art methods have been used in the forestry industry for these purposes previously. A deep-learning framework called Faster Region-based Convolutional Neural Network (Faster R-CNN) [33] and its predecessor R-CNN [34] have been within the top performing deep-learning models for object detection in recent years [33]. In Faster R-CNN, the input image is given to a convolutional network which outputs a convolutional feature map. A region proposal network (RPN) is then used to predict regions which are likely to contain the objects (stumps in our case). A ROI pooling layer is used to reshape the region into bounding boxes and classify them.

## 2. Materials and Methods

**Figure 1:**
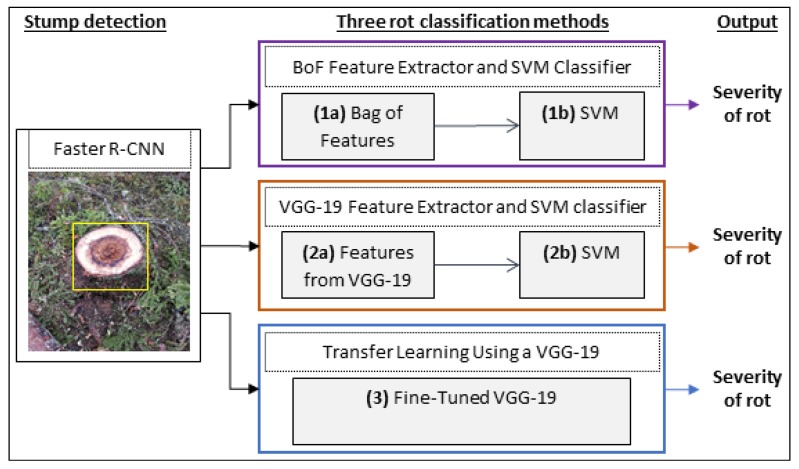
Stumps are detected using Faster-RCNN and sent to the rot classification, where three different methods have been evaluated. Two different SVMs (1b and 2b) are trained and tested sequentially based on the features extracted from Bag of Features (1a) and the FC-7 layer of an VGG-19 (2a) respectively. The third method is using a fine-tuned VGG-19 to both extract features and classify the rot. Each method outputs the severity of rot in the detected stump into one of three classes: rot = 0%, 0% < rot < 50% and rot ≥ 50%.

To simulate images taken from a harvester head or crane boom, images of stumps from newly harvested trees were taken as described in Section 2.1. To detect stumps in the images a Faster R-CNN network was used as described in Section 2.2. The extracted stump was then fed into a rot classification system which uses three different methods, both conventional machine-learning and deep-learning methods, to classify the severity of rot as further described in Section 2.3. Rot was classified into 3 classes corresponding to the quality requirements stipulated by industry, where no rot (0%) is permissible in saw timber, up to 50% rot is allowed in prime grade pulpwood, while logs containing more than 50% rot are further downgraded. Figure 1 shows a schematic workflow of the implemented algorithm.

### 2.1. Image Collection

**Figure 2:**
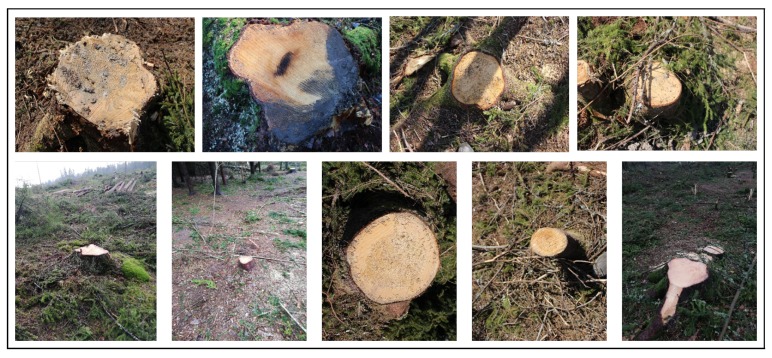
Sample of images in the image collection with different distances to the stumps, occlusions, and lighting conditions.

For image acquisition we used a Canon EOS 100D camera. Images were collected at different post-harvest sites near to the town of Ås, in south-eastern Norway. Since the final aim is to use the trained system on the harvester to detect and classify stumps and RBR in real time, the freshness of the cut stumps is important since an oxidation process changes the color of the stump surface. Therefore, all the images in the data set are taken within a few days after the harvesting operation. Images were taken at different distances and angles toward the stumps, various times of day with different lighting conditions and varied degree of occlusion to simulate possible settings of a camera on the harvester crane and real working environment (See Figure 2). In total 1000 images were acquired and divided to three sets of stumps; 502 images without evidence of RBR, 197 with RBR ratio <50% and 301 with RBR ratio >50%. The rot area ratio (RBR ratio) was computed pixelwise using manually segmented stumps.

### 2.2. Stumps Detection Using Faster R-CNN

In this work we trained a stump detector using Faster R-CNN [33] from MATLAB’s Computer Vision System Toolbox following these steps:

1. Since the image size directly affect the training time, the original image size (3456 × 5184) was reduced to 300 × 400 pixels. This was a tradeoff between speed and accuracy. The dataset was divided into training and testing sets such that 80% of all images were used for training the Faster R-CNN and the rest for evaluating the result.
2. To create a convolutional neural network (CNN) the input, middle, and final layers are stacked on top of each other. The size of the input layer must be similar in size to the smallest object in the data set. In this work we chose 32 × 32 pixels. The middle layers include two convolutional layers with 32 filters of size 3 × 3, two rectified linear units (ReLU), and a pooling layer. The final layers of the CNN contain a fully connected layer with 64 output neurons, a ReLU layer, a SoftMax layer, another fully connected layer with two classes (stumps and background) and a classification layer.
3. To train the Faster R-CNN we used the “trainFasterRcnnObjectDetector” function from the Computer Vision Toolbox in MATLAB. It trains the detector in four steps [33]. In the first two steps, it trains the region proposal and detection networks and in the final two steps it combines the trained networks (from the first two steps) to create a single network for detection. Each training step has different training configurations. In this work we set all the training parameters equally for all four steps (mini-batch size = 1, solver = sgdm), except the learning rate and maximum number of epochs. The learning rate was set to a lower value in the final two steps (1 × 10${}^{-6}$ compared to 1 × 10${}^{-5}$ for the first two), as they are fine-tuning steps and the network weights can be modified more slowly. The number of epochs were reduced from 20 to 10 in the last training step to reduce training time.

### 2.3. Rot Classification

Extracting features from regions of interest and training classifiers is a general approach for object classification in computer vision. Selecting a proper set of features has an essential role in classification accuracy. In similar works for wood defect detection [18,35] researchers have used several features based on color and texture of RBR. These features are selected randomly based on the environmental conditions. In this work we compare three approaches with different methods of feature extraction and classification for the task of rot classification:

1. **BoF Feature Extractor and SVM Classifier**. An alternative technique for extracting features in object classification tasks is using Bag of Features (Bag of Visual Words) [36] which is adapted from the natural language processing field. In this method first a vocabulary of SURF [37] features is constructed to represent images. These features are grouped into *k* clusters, using k-means, where each cluster center represents a feature. In this work *k* was set to 500. The extracted features were then used to train a multi-class SVM classifier for detecting existence of RBR on stumps.
2. **A VGG-19 CNN-based Feature Extractor and SVM Classifier**. In this approach, instead of selecting features manually, we extract them from a fully connected layer of a CNN. After that, a multi-class SVM classifier is trained using these features. For this purpose, we used the FC-7 layer of a VGG-19 [38] convolutional network in MATLAB. The VGG-19 network is pre-trained on more than one million images from the ImageNet dataset, 47 layers deep and can classify objects into 1000 classes. Therefore, the network is well suited to extract a set of effective features from a region of interest for classification. In this convolutional network, the first layers learn filters for capturing general features such as edge and blob. These features are processed further and combined in deeper layers which make them more suitable for the task of recognition. Basically, any layer in the convolutional network can be selected to represent features; however deeper layers contain richer features. Therefore, we selected the last fully connected layer (FC-7) before the classification layer. This layer presents 4096 features for each input object.
3. **Transfer Learning Using a VGG-19 CNN**. In this approach we used the same VGG-19 network used above but applied fine-tuning to perform feature extraction and rot classification. Fine-tuning a pre-trained network with transfer learning is much faster and easier than training a network from scratch. This enables transferring learned features to a new classification task, using a smaller number of images for training. To fit the VGG-19 CNN to our specific classification task we replaced the last three layers (fully connected, SoftMax, and classification) with five new and then re-trained the whole network. The new layers were: a fully connected layer with 64 output neurons; a ReLU layer with the aim of adding non-linearity to the network; a fully connected layer with three output neurons (representing the three classes of rot severity); a SoftMax layer; and a classification layer. An important parameter in a CNN network is the learning rate which controls how much a network change during the training process. As the network is pre-trained, we kept the learning rates in the original layers of the network unchanged and instead boosted the learning rate of the last fully connected layer. This way earlier layers do not change much in the training process while new layers learn the weights faster. The network was trained with batch size = 16, initial learning rate = 1 × 10${}^{-4}$ and maximum number of epochs = 300.

The input to the classifiers were the correctly detected stumps (True Positives) from the Faster R-CNN network (Section 2.2). For comparison we also did tests where we used the manually labeled stumps (cropped to the stump region). Each classifier was trained and evaluated on two different datasets; one with two classes of stumps with RBR and without RBR, and one with three classes: RBR = 0%, 0% < RBR < 50%, and RBR ≥ 50%. To prevent bias toward any class, we used the same number of training images for each class, in this case 160 images (the total number of images in the class with rot ≥ 50%). The same training and testing samples were used for all three classifiers to be able to compare the performance.

We re-sized the detected stumps to the original size and then fed them to the classifiers. This is beneficial for BoF+SVM classifier since it gets richer features. For the other two classifiers, the VGG-19 network re-sized the input image to [224 224].

### 2.4. Segmentation Quality

**Figure 3:**
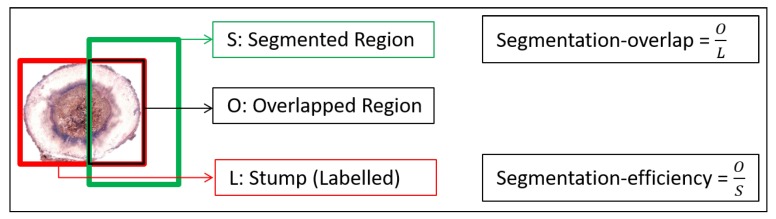
Segmentation quality using Segmentation-overlap and segmentation-efficiency.

Segmentation in an object detection approach means dividing the image into foreground (object of interest) and background. Therefore, it is essential to evaluate the quality of the segmentation process. In this work, the segmentation quality shows how well the area of the detected object matches to the manually labeled object. We used two measures to estimate the segmentation quality: segmentation-overlap and segmentation-efficiency (see Figure 3) [39,40]. Segmentation-overlap is defined as the ratio between the overlapped region (*O*) and the manually labeled region (*L*), where overlap is the common area between the segmented region (*S*) and the manually labeled region (*L*). It demonstrates which percentage of the labeled area is actually segmented. For example, if segmentation-overlap is 0.6, then 60 % of the stump is also segmented. Segmentation-efficiency is defined as the ratio between the overlapped region (*O*) and the segmented area (*S*). It indicates how large part of the segmented area contains the actual object. For example, if segmentation-efficiency is 0.9, then 10% of the segmented area covers the background and 90% covers the stump.

A low segmentation-overlap and a high segmentation-efficiency score indicates that the detected region covers a small area of the actual object; however, this region is a large part of the detected area. On the other hand, a high segmentation-overlap and low segmentation-efficiency means that a large part of the object is covered by the segmented region, while a large part of the segmented region is the background. The measure of segmentation quality (sq) is the average of segmentation-overlap and segmentation-efficiency:

(1) $$sq=\frac{\frac{O}{L}+\frac{O}{S}}{2},$$

and it is within the range [0,1]. In this method, if both segmentation-overlap and segmentation-efficiency are 1, then we achieve the best segmentation quality. It shows that the segmented region (*S*) completely overlapped with the labeled region (*L*).

## 3. Results

### 3.1. Stump Detection

Since the aim of stump detection is to segment stumps with and without RBR in images, the proposed approach using Faster R-CNN algorithm was trained on 799 images of stumps in both classes. The trained detector was then tested on the remaining 201 images. If segmentation quality sq of the detected area is higher than 0.5 (see Section 2.4, Equation (1)), then the detected area is considered to be true positive (TP), otherwise the segmented area is counted as false positive (FP). Also, if any stump was not detected then it was false negative (FN). The proposed method could correctly detect 162 stumps; however, it missed 39 and falsely segmented 9. This resulted in a precision rate of 95% and 80% recall, meaning that that 95% of all segmented areas correctly represented a stump, but 20% of stumps were not detected.

### 3.2. Rot Classification

To determine how well the classifiers worked, we first evaluated them with manually segmented stumps. For simplicity we call classification approaches by their abbreviations, **BS**: BoF and SVM classifier, **VS**: VGG19 and SVM and **VF**: Fine-Tuned VGG19. As it is shown in Table 1, for classifying stumps into two classes (stumps with and without RBR) BS could correctly classify 63% of healthy stumps and 74.4% of stumps with RBR, VS increased the performance of healthy stump classification to 86.4%, while classification performance for stumps with RBR is reduced to 71.4%. VF approach achieved the highest performance with 91.2% accuracy for healthy stumps and 90.8% for stumps with RBR. Classifying stumps into three classes (rot = 0%, 0% < rot < 50%, rot ≥ 50%) decreased the classification performance, see Table 2. Similarly, BS approach resulted in the lowest accuracy and VS increased classification performance except for the third class. VF reached the highest performance for all three classes: 81.5% for the class without rot, 83.6% for the second class, and 78.3% for the class with rot ≥ 50%. Results demonstrate that VF approach achieved the highest performance for classifying stumps to either two or three classes.

To assess performance of the whole application (both stump detection and rot classification), we used the correctly segmented stumps (TP) from the stump detector as input for the classifiers. Firstly, we measured classification performance for two classes of stumps. As it is shown in Table 3, the BS approach resulted in the lowest performance. The VS approach had the best performance on correctly classifying unhealthy stumps while being much worse on healthy ones. The VF approach attained the highest performance, 83.5%, for healthy stumps, while performance of stumps with RBR was reduced to 77.5%, which is slightly lower than for VS. Accuracy evaluation of classification approaches for three classes of stumps (see Table 4) demonstrated that similar to previous evaluations, BS approach had the poorest performance. Classification accuracy using VS approach increased for the first two classes but decreased for stumps with rot > 50%. VF attained the highest classification accuracy for all three classes: 79.4%, 72.4%, 74.1% respectively. Generally, the performance of the classification approaches is higher when using two classes compared to three.

## 4. Discussion

This research work proposed a novel method for stump detection and RBR classification of Norway spruce in post-harvest sites with the aim of acquiring stump information. In recent years only a few studies such as [17,18] with acceptable performance have been dedicated to stump detection and classification in forestry environment using computer vision techniques. Other work in detecting defects in wooden boards and tree logs was mostly conducted in environments with controlled conditions and are not directly comparable [22,23,26,28]. Using image analysis and computer vision techniques in real working environment is a relatively new topic in this field, therefore there are a limited number of results to make comparisons with. We compare our results with Puliti, et al. [18] achievements, due to the similar objectives of detecting and classifying stumps in a forestry environment.

The proposed stump detection algorithm using Faster R-CNN achieved a precision rate of 95% and recall 80%. These results are obtained using stumps in different lighting conditions, partially damaged or occluded and clearly visible stumps at various distances and sizes (see Figure 2). These results are comparable to those that were reported by Puliti, et al. [18], where they achieved detection accuracy of 67.9% to 79.9% for all types of stumps and for visible and not damaged stumps, respectively.

To determine how well our classification approaches works for classifying stumps to either two or three classes, we first tested them with manually segmented stumps. Results revealed that the conventional classification strategy, BoF+SVM, had the poorest performance. A reason for this could be a limited number of distinguishing features between stumps with and without RBR. An example of these features can be color and texture; however, these features can easily present difficulties in presence of shadows or occlusions by needles, branches, and sawdust. Extracting features from the last fully connected layer of VGG19, and using them to train an SVM classifier, increased the classification performance. One explanation can be that since VGG19 is trained on millions of images, it is trained to extract more efficient features which results in escalating the performance. Using fine-tuned VGG19 for classification of stumps resulted in the best performance in comparison to other approaches. It shows that retraining the CNN with new images makes it more capable in extracting effective features which fits well with the trained dataset. Assessing all the results confirms the repetition of this routine and revealed that transfer learning using VGG19 CNN attained the highest classification accuracy in all the testing cases. Testing the proposed algorithm on the correct (TP) outputs of the stump detector, fine-tuned VGG19 achieved classification accuracy of 83.5% and 77.5% for stumps without RBR and with RBR, respectively. Comparing these results with the classification accuracy achieved by Puliti, et al. [18], 74.5% and 82.5% for stumps without RBR and with RBR, shows that while classification accuracy for stumps without RBR is increased, it is reduced for the class of stumps with RBR; however the overall accuracy is slightly increased.

Moreover, comparing classification results with defect detection accuracies achieved by Ruz et al. [26], 95% accuracy with only 6% of FP and [23], with 91% of correct classification, demonstrate that they achieved higher performance. However, it is essential to address that these results are achieved by applying classification algorithm on clear boards of wood in a controlled environment where illumination conditions are manually adjusted and it is the same for all the images, while we achieved these results in a dynamic and uncontrolled forest environment.

**Figure 4:**
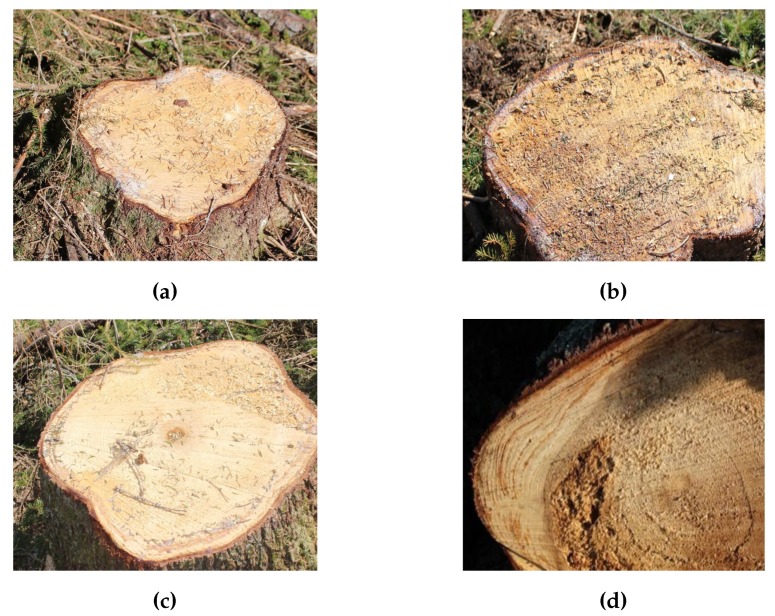
Examples of misclassification. Detected stumps were labeled as: (**a**) rot = 0%, (**b**) rot = 0%, (**c**) rot < 50%, and (**d**) rot > 50%, but were classified as: (**a**) rot > 50%, (**b**) rot > 50%, (**c**) rot > 50%, and (**d**) rot = 0%.

Comparing classification accuracies demonstrates that classifying stumps to three classes attained lower performance than two classes. One argument can be that 160 training images in each class is not enough to distinguish differences between the classes, especially for borderline cases. For example, if the rot value is 49% then there is a high probability that the stump is classified in the third class (≥50%) and similar for stumps with very little rot. Since distinguishing the amount of rot in such cases is difficult, flexible borders might be defined between classes. Moreover, as illustrated in Figure 4, presence of sawdust, needles, branches, and shadows could result in misclassifying the detected stump.

**Figure 5:**
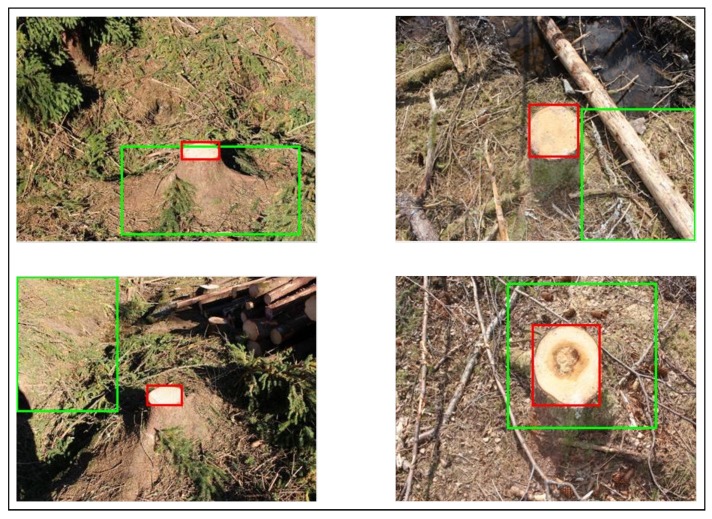
Comparing results of stump detector with the manually segmented stump. Red and green bounding boxes demonstrate manually segmented stump and result of stump detector, respectively.

Compared to manually segmented stumps, the classification accuracy decreased when using the outputs of the stump detector. Comparing outputs of the stump detector with manually segmented stumps revealed that in some cases the detected areas with sq ≥ 0.5 (See Section 2.4) do not perfectly match with the manually segmented stumps. As it is shown in Figure 5, parts of background (stump trunk, needles and branches) are included in the detected area, while manually segmented areas represent only the stump surface. Since classification approaches are trained based on manually segmented stumps, these additional parts of the background could be the main cause of reduced accuracy. This complication can be reduced by increasing the sq threshold; however, it might reduce the stump detection accuracy. This also explains the difference between our results and what Puliti, et al. [18] achieved in rot classification. They used stump segments which include both the stump and parts of the background for training and testing, which helps the classifier to learn about the background, while in our work we only used the stump surface for training and then testing it on segments that include parts of background. This analysis shows the need for including background areas in the training sets or improving the stump detector approach such that it only outputs the stumps surface.

The novelty of this research work is in using deep-learning approaches in the forestry industry for the purpose of stump detection and rot classification. To confirm the achieved performance and improve proposed approaches, it would be necessary to assess them in real working environments by mounting a camera on the crane of a harvester. However, image quality and detection success might be greatly improved by fitting a camera to image the butt end of the stem once harvested, although there remain several technical challenges in getting this to function as intended [41]. The advantages of a camera mounted inside the harvesting head would be the relative clarity of the log face, as well as a fixed distance and angle to the log.

The system reported in this study can be used for autonomously categorizing harvested tree stumps to healthy and RBR infected ones. This is done offline, but since it only took 650 milliseconds on average to detect a stump and 160 milliseconds to classify it using a fine-tuned VGG-19, it is quite possible to do this in real time for assisting a machine operator to determine the level of rot while harvesting a tree. This shows the potential for reducing the mental work load of machine operators while providing a more stable and reliable source of information. Combining data from the proposed algorithm and GPS, it would be possible to coarsely localize stumps with root and butt-rot to generate an RBR distribution map of the area. Such a map could be used in planning regeneration strategies in reducing the spread of RBR.

## 5. Conclusions and Future Work

In this research, we used deep-learning CNN-based approaches in a novel application, the detection and classification of RBR in a forest environment. Although the results were acceptable, the proposed algorithm requires further enhancement and validation, possibly combined with an improved placement of the camera. One thing to test in future work is to switch Faster R-CNN with YOLOv3 [42], which shows higher performance in object detection tasks. Additionally, to improve the classification accuracy, it would be possible to adopt residual learning framework [43] which is 8 times deeper than VGG nets, but has lower complexity.

## Figures and Tables

**Table:** **(a)** BoF + SVM

-	No Rot	Rot	Classification Accuracy
No Rot	65	38	63.0%
Rot	25	73	74.4%

**Table:** **(b)** VGG19 + SVM

-	No Rot	Rot	Classification Accuracy
No Rot	89	14	86.4%
Rot	29	70	71.4%

**Table:** **(c)** VGG19 Fine-Tuned

-	No Rot	Rot	Classification Accuracy
No Rot	94	9	91.2%
Rot	9	89	90.8%

**Table:** **(a)** BoF + SVM

-	Rot = 0%	0% < Rot < 50%	Rot ≥ 50%	Classification Accuracy
Rot = 0%	61	7	35	59.2%
0% < Rot < 50%	7	26	28	42.6%
Rot ≥ 50%	8	4	25	67.5%

**Table:** **(b)** VGG19 + SVM

-	Rot = 0%	0% < Rot < 50%	Rot ≥ 50%	Classification Accuracy
Rot = 0%	67	16	20	65.0%
0% < Rot < 50%	5	47	9	77.0%
Rot ≥ 50%	5	8	24	64.8%

**Table:** **(c)** VGG19 Fine-Tuned

-	Rot = 0%	0% < Rot < 50%	Rot ≥ 50%	Classification Accuracy
Rot = 0%	84	12	7	81.5%
0% < Rot < 50%	6	51	4	83.6%
Rot ≥ 50%	3	5	29	78.3%

**Table:** **(a)** BoF + SVM

-	No Rot	Rot	Classification Accuracy
No Rot	19	54	26%
Rot	27	62	69.6%

**Table:** **(b)** VGG19 + SVM

-	No Rot	Rot	Classification Accuracy
No Rot	36	37	49.3%
Rot	9	80	89.8%

**Table:** **(c)** VGG19 Fine-Tuned

-	No Rot	Rot	Classification Accuracy
No Rot	61	12	83.5%
Rot	20	69	77.5%

**Table:** **(a)** BoF + SVM

-	Rot = 0%	0% < Rot < 50%	Rot ≥ 50%	Classification Accuracy
Rot = 0%	12	9	52	16.4%
0% < Rot < 50%	12	15	31	25.8%
Rot ≥ 50%	2	5	24	77.4%

**Table:** **(b)** VGG19 + SVM

-	Rot = 0%	0% < Rot < 50%	Rot ≥ 50%	Classification Accuracy
Rot = 0%	32	24	17	43.8%
0% < Rot < 50%	14	33	11	56.8%
Rot ≥ 50%	2	14	15	48.3%

**Table:** **(c)** VGG19 Fine-Tuned

-	Rot = 0%	0% < Rot < 50%	Rot ≥ 50%	Classification Accuracy
Rot = 0%	58	11	4	79.4%
0% < Rot < 50%	4	42	12	72.4%
Rot ≥ 50%	1	7	23	74.1%
